# Elephant in the room - Family members´ perspectives on advance care planning

**DOI:** 10.1080/02813432.2020.1842966

**Published:** 2020-11-11

**Authors:** Lisa Kastbom, Marit Karlsson, Magnus Falk, Anna Milberg

**Affiliations:** a Department of Health, Medicine and Caring Sciences, Linköping University, Linköping, Sweden; b Primary Health Care Center in Ljungsbro, Linköping University, Linköping, Sweden; c Department of Advanced Home Care in Linköping, Linköping University, Linköping, Sweden; d Department of Biomedical and Clinical Sciences, Linköping University, Linköping, Sweden; e Primary Health Care Center in Kärna, Linköping University, Linköping, Sweden; f Department of Advanced Home Care in Norrköping, Linköping University, Linköping, Sweden

**Keywords:** Advance care planning, education, family member, family practice, nursing homes, qualitative research

## Abstract

**Objective:**

To explore family members’ experiences of advance care planning in nursing homes.

**Design:**

Individual interviews. Thematic analysis.

**Setting:**

Four nursing homes in Sweden.

**Subjects:**

Eighteen family members of deceased nursing home patients.

**Main outcome measures:**

Family members’ experiences of advance care planning in nursing homes.

**Results:**

Family members’ experiences of advance care planning in a nursing home context involved five themes: *Elephant in the room*, comprising end-of-life issues being difficult to talk about; *Also silent understanding*, e.g. patient’s preferences explicitly communicated, but also implicitly conveyed. In some cases family members had a sense of the patient’s wishes although preferences had not been communicated openly; *Significance of small details*, e.g. family members perceive everyday details as symbols of staff commitment; *Invisible physician, supporting nurse*, e.g. nurse being a gatekeeper, providing a first line assessment in the physician’s absence; and *Feeling of guilt*, e.g. family members wish to participate in decisions regarding direction of care and treatment limits, and need guidance in the decisions.

**Conclusion:**

Our study stresses the significance of staff involving the patient and family members in the advance care planning process in nursing homes, thereby adapting the care in line with patient’s wishes, and for the patient to share these preferences with family members. Education in communication related to the subject may be important to shape advance care planning.

**Key points**
- Knowledge on advance care planning (ACP) in a nursing home (NH) context from the perspective of family members is limited.
- Role of the nurse in ACP is seen as central, whereas physician involvement is often perceived to be lacking.
- Significance of small details, perceive to symbolize staff competence and respect for patient autonomy.
- To limit family members’ feeling of guilt, communicating end-of-life issues is important in order to align ACP with patient preferences.

## Introduction

Advance care planning (ACP) is generally seen as a central aspect of care for patients with severe illness [1–3] and there are indications that ACPs may influence the quality of care positively [1,4–6]. Patients in nursing homes (NHs) are often severely ill, and they, as well as their family members, seem to have a positive attitude to ACPs in general [7–10], and view ACP as an opportunity for the patient to communicate preferences and share them with the family members [7,9,10].

Family members are often an important part of the ACP process [6,8,11,12], e.g. to support patients during ACP, to help patients to communicate their preferences for end-of-life care to the nurse or physician, and to represent patients’ thoughts when they no longer can communicate preferences or make decisions on their own.

Involvement of family members could enhance the quality of ACP and benefit the NH patient’s participation in ACP [6]. However, family members are often worried about making wrong decisions when asked concerning direction of care and treatment limits [13]. Also nurses and physicians experience challenges in these situations. In a previous study we found that nurses and physicians are afraid of being accused of maleficence, e.g. when initiating ACP or deciding the level of care in an acute situation [12]. In Sweden, only physicians, not nurses or the family members, have the authority to make decisions regarding life-sustaining care and restrictions, possibly based on patient preferences [14]. When decisions regarding end-of-life care must be made, and the preferences of the patient are not known, complicated situations and ethical dilemmas can arise [12,13]. The value of ACP when caring for people living in NHs has been described as ‘a neglected research topic’ [15]. Despite the fact that family members are important in the process of ACP [6,11–13], there is more to investigate regarding ACP in NHs from the perspective of family members, for example regarding their role in ACP and their experiences in decisions concerning treatment limits. Half of the annual deaths in Sweden take place in a NH [16], which makes it especially important to study ACP in this context. Therefore, the aim of this study was to explore family members’ experiences of ACP in NHs in Sweden.

## Material and methods

### Participants

A family member was defined as a person who was specified, in the patient’s medical record, to be contacted concerning patient issues. This person did not necessarily need to be a relative of the patient. Inclusion criteria were: being a family member of a patient who lived in a NH at the end of life, being an adult and accepting the interview to be recorded. Exclusion criteria were: inability to understand and/or speak Swedish sufficiently, or cognitive impairment. Four NHs were included in the study.

Participants were recruited through purposeful sampling [17]. About four to eight weeks after the death of the NH patient, possible informants who met the inclusion criteria of the study, were asked by a nurse working at the NH if they agreed to be contacted by the research team for more information about the study. Usually, this contact by the nurse was made during a so-called ‘bereavement follow-up’. Participant inclusion continued until redundancy appeared [17].

### Interviews

Individual interviews were conducted about three months after the death of the patient. All the interviews were performed by a researcher with experience of working as a general practitioner (GP), where dealing with NH patients is an essential part of daily practice. None of the researchers worked at any of the NHs from which the informants were recruited.

An interview guide [17] was developed by the research group. This interview guide consisted of open questions concerning, for example, the shape of end-of-life care, including ACP and treatment limits. The interviews were conducted in 2018–2019 and were digitally recorded and transcribed verbatim.

### Analysis

As the purpose of this study was to search for themes/patterns of family members’ experiences of the topic, thematic analysis [18] was considered to be suitable. An inductive, latent approach [18] was chosen. The interviews were analysed using the following steps: (1) familiarizing with the data, (2) generating initial codes, (3) searching for themes, (4) reviewing themes, (5) defining and naming themes and (6) producing the report [18].

The initial coding was mainly done by the researcher who performed the interviews and the main supervisor. The results of the initial coding and different views and meanings were then discussed by the whole research group until agreement was reached, as part of validating the findings [17].

The research team consisted of two GPs with experience of working with patients living in NHs, and two senior consultants in geriatrics and palliative care. The different experiences and backgrounds strengthened the reflexivity process [17]. All four researchers had experience in qualitative research.

## Results

**Figure 1.**
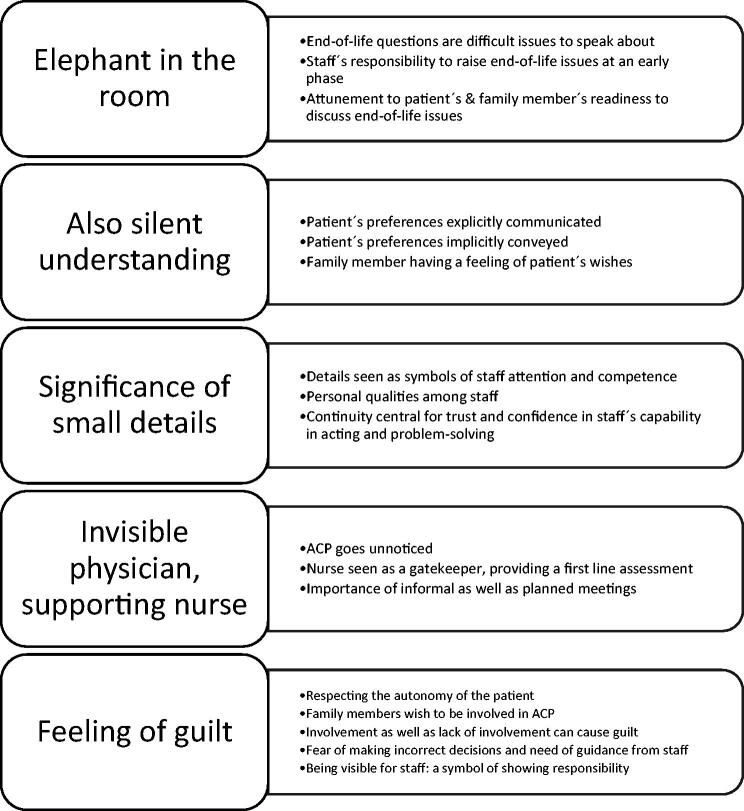
Final thematic map illustrating the main themes describing the ACP process in a nursing home context from the perspective of family members.

In total, 18 informants were interviewed. There was broad variation in terms of age, gender, type of relation to the deceased nursing home patient (Table 1) as well as patient factors such as age, gender, time from moving to the NH until death, co-morbidities and presence of cognitive impairment (Table 2). When analysing the data, five themes describing family members’ experiences of ACP in NHs were seen (Figure 1).

**Table 1.** Background characteristics of the 18 participants.

Age mean (range)	62 years (39–75)
Gender	
Men (% (n))	39% (7)
Women (% (n))	61% (11)
Relationship to deceased patient	
Son/daughter (% (n))	67% (12)
Husband/wife (% (n))	11% (2)
Brother/sister (% (n))	5,5% (1)
Grandson/granddaughter (% (n))	5,5% (1)
Niece/nephew (% (n))	11% (2)
Employment	
Working person (% (n))	39% (7)
Pensioner (% (n))	61% (11)
Place for interview	
Health center (% (n))	61% (11)
Home/office of participant (% (n))	11% (2)
By telephone (% (n))	28% (5)
Time from death to interview mean (range)	93 days (82–131)

**Table 2.** Background characteristics of deceased nursing home patients.

Age by death, mean (range)	88 years (69–99)
Gender	
Men (% (n))	28% (5)
Women (% (n))	72% (13)
Place of death	
Nursing home (% (n))	89% (16)
Hospital (% (n))	11% (2)
Time from moving to NH till death, mean (range)	32 months (<1–98)
Presence of dementia/MCI diagnosis (y/n)	44% (8) / 56% (10)
Numbers of nursing homes participating (n)	4
Nursing home(s), rural location (n)	1
Nursing homes, urban location (n)	3

### Elephant in the room

Although most family members had realised that the health of their relative was deteriorating and death was approaching, end-of-life issues had been difficult to talk about with both the patient and staff.

Some informants expressed thoughts about the patient being afraid of talking about end-of-life and death with the family members due to the risk of causing them harm and sadness.

In her [the patient/mom] world, I was a child. Her child. She wished to spare me a little … I think she wanted them to avoid speaking about it [end-of-life issues] when I was there.

(Daughter of 88-year-old woman.)

In addition, family members had noticed that also staff showed such reluctance to talk about these topics to avoid contributing with worry and sadness to the patient and family members.

The informants emphasised the significance of staff raising these questions with both the patient and family members, in order for the staff to know the preferences and views of the patient and his or her family members. Communicating end-of-life issues, death and dying with the patient at an early phase, while the patient was still cognitively intact, was preferable, as doing so made it possible to provide future care in line with the patient’s preferences, despite in the case of for example cognitive impairment. However, according to the informants, these discussions often did not happen.

Maybe, it’s not the first thing to do, but sometimes during the trajectory … We all know that it will come, but no one [patient, family members or staff] wants to talk about it.

(Granddaughter of 95-year-old woman.)

It’s difficult to raise the question in an acute situation. Mm. If you raise the question directly, the first week … when it [end-of-life/death] is not that impending … then it’s maybe easier to handle … the difficult issues directly, before panic occurs.

(Daughter of 81-year-old woman.)

Although it was considered important to have these discussions at an early phase, the informants underlined the importance of staff’s attunement to the patient’s and family members’ readiness to discuss end-of-life issues and, if they were reluctant, these questions sometimes should be postponed and raised later.

### Also silent understanding

An understanding of the patient’s preferences could be based on explicit communication in the moment, as well as previously communicated thoughts, sometimes based on the patient’s experiences of the death of others.

… Mum and I had talked about it [wishes regarding end-of-life] before she moved to the nursing home and before she got the dementia … so I was confident about her [end-of-life] wishes.

(Son of 88-year-old woman.)

He [dad] was there for a long time, connected to those things [medical equipment]. And she [mum] didn’t want it to be that way for her, she said.

(Son of 99-year-old woman.)

Sometimes the patient’s preferences had been implicitly conveyed. That is, although the patient’s preferences were not communicated clearly, the family felt confident they knew the wishes of the patient. In order to achieve this, the relationship between the patient and the family member had to be close. Silent understanding of the patient’s wishes, i.e. wishes regarding end-of-life and dying were not communicated, either explicitly or implicitly, but family members had a sense of the patient’s wishes. Silent understanding was based on knowledge of the patient’s life, experiences and values, as well as the family members’ ability to apply this knowledge in the situation.

I can’t remember [that we spoke openly about her end-of-life wishes]. I think I had a feeling about what she wanted.

(Niece of 97-year-old woman.)

### Significance of small details

The informants frequently mentioned positive as well as negative details, or small things, such as staff doing more than just the ordinary routine tasks (positive) or the patient’s room not being tidy (negative). These positive or negative aspects of care had been of importance in how the informants had experienced the present, on-going care of the patient. In addition, these details were of symbolic importance regarding staff engagement, competence and commitment, and how the patient was seen and treated overall, also when being at the end-of-life. Furthermore, these details were experienced as long-term indicators of staff having the capability of resolving forthcoming challenges in the care of the patient, and therefore reflected trust in staff following the ACP and respecting patient’s preferences and autonomy. In contrast, experiences of negative details, or small things, were connected with expectations of staff not being engaged, competent and committed, and the risk of the patient not being treated in line with his or her preferences in the future. Although the negative details were not always connected to negative consequences for the patient/family members at the time they happened, and were sometimes more like nuisances (e.g. the extra spare morphine package not ordered as planned by the staff, even though it was not needed at the moment since the patient did not have any pain), they were experienced as negative indicators regarding the future care of the patient.

A girl who was fantastic with the old ones and did a lot with them … She made them happy every day with different activities and. Mm. She organised a Nobel dinner for them. And a party after Christmas with bags of sweets … That little extra … changed watch batteries, helped them with their hearing aids, and such things that they normally don’t do.

(Niece of 97-year-old woman.)

The management of medicines didn’t always work well … I had to go and search for medicine deliveries … They [staff] simply didn’t know where the medicine was stored.

(Nephew of 97-year-old woman.)

Informants valued staff being personal and showing engagement and commitment, as mentioned above. This was more likely when there was a stable situation at the NH with staff continuity. As a consequence of being satisfied with the positive small things that mattered and trusting the staff, family members could focus on socializing with their relative when visiting the NH, and felt confident regarding the future care of the patient.

### Invisible physician, supporting nurse

The informants described having little contact with the physician. In contrast to rich descriptions of the nurse, the informants’ descriptions of the physician at the NH, and the relations with him or her, were limited. It was notable that ACPs and formal meetings with the physician preceding an ACP document, often passed unnoticed. According to the informants, the meetings at the NH were mostly without physician participation and several informants could not remember being offered a meeting with the physician, nor having participated in an ACP meeting.

We only saw the physician once or twice for the whole period [NH patient living at the NH for 14 months]. I cannot say anything about him … How he was …

(Daughter of 96-year-old woman.)

The informants viewed the nurse, not the physician, as the central person in the care of the patient at a NH, and in the interviews the nurse was often generously described. The nurse could be regarded as a gatekeeper, providing a first line assessment, reporting to, or consulting the physician if necessary. Given that the nurse was in attendance, available, responsible and knowledgeable, and that the family members had developed trust in him or her, some expressed little need to see a physician. Some family members had been offered a meeting with the physician, but declined, as they were satisfied with the communication with the nurse.

I had a good relationship with the contact person as well as the nurse. And they often asked me if I wanted to see the physician. But then I said, I said nearly every time

`No, there’s no need to do that. You and I have a very good relationship and if you want to check something medically, you’re welcome to talk to the physician…

(Brother of 69-year-old woman.)

The informants appreciated planned meetings with the staff. Informal meetings, with the nurse, contact person, or other staff at the NH, were also considered to be of importance. These informal meetings symbolised engaged and committed staff.

When I looked for him [the nurse] he always had time for me when I caught him in the corridor … And he always said I could contact him whenever I needed, and also afterwards if I wished to have a support dialogue or something like that.

(Daughter of 88-year-old woman.)

### Feeling of guilt

Family members stressed the importance of respecting the patient’s preferences, i.e. the importance of respecting the autonomy of the patient was central. The informants appreciated being involved in ACP and being able to make plans proactively. However, involvement, as well as lack of involvement in ACP, could also contribute to family members feelings of guilt. For example, family members could blame themselves for not having taken the initiative to talk about questions regarding end-of-life while the patient was still capable of communicating his or her thoughts.

Although family members wished to participate in decisions regarding the direction of care and treatment limits, they highlighted a need for guidance in these decisions. When family members had found that they were supposed to make difficult decisions without support and guidance from staff, a fear of making incorrect decisions emerged.

… Who’s the one to make the decision [about treatment limits]? It was so difficult for me to make the decision, but I didn’t know who else would make it. Maybe, it’s me, but I also wanted health care staff to tell me that this would be the best in this case … What if I say the wrong thing…

(Daughter of 87-year-old woman.)

Presence at the NH as a family member could be seen as a symbol of showing responsibility in taking part of the care of the patient, and was also important for building good relations with staff, according to the informants. In contrast, not attending and not taking the initiative to contact staff could be interpreted as not showing responsibility, which could contribute to guilt. When the informants found that the staff were the ones who were responsible for making challenging and difficult decisions, even if the family members were involved in the ACP process, the family members did not feel they had to shoulder the entire responsibility; thus they found that the health care staff was balancing the burden for them.

I think we, being family members, were not visible at the NH. We had our contact with grandma, not that much with staff actually … Maybe they thought that we didn’t want to participate in the care of grandma…

(Granddaughter of 95-year-old woman.)

[About ACP] *We talked a lot about this … that they would make it easier for him … ACP at the NH was very good. Because they spoke about making his situation, the time he had left, as comfortable as possible.*

(Daughter of 90-year-old man.)

## Discussion

The present study showed that family members found that staff often refrain from communicating about the patient’s deteriorating health. This is in accordance with previous research, where studies have indicated that ACPs are not practiced as widely as recommended [19]. Even though patients often want to communicate with their physician about their wishes for future care [5,20–22], the vast majority of patients with life-threatening diseases and older individuals have never discussed such end-of-life care issues with their physician [4,22,23]. Sharp et al. showed that there is a great discrepancy between the majority of old patients who wish to discuss end-of-life issues, and the minority of this patient group that actually get this opportunity [22]. Our study stressed that it is not only staff who refrain from communicating about the patient’s approaching death with the family members. Also the patients can be reluctant to communicate about this with their family members. Such lack of information may hinder the family members from engaging in the care and ACP of the patient, and may contribute to guilt. It seems important that staff actively invite family members, where appropriate according to the patient’s wishes, to participate in the care and in the ACP also facilitate communication about such matters.

In this study, family members felt that end-of-life questions should be raised by staff and that this preferably should be at an early phase, when the patient is still competent, in order to explore the patient’s wishes and values, and the ACP can be adapted in coherence with the patient’s autonomy. However, previous studies indicate different views from the perspective of older patients regarding talking about death and dying. In a meta-synthesis, Ke et al. showed that although many older patients wish to communicate end-of-life questions, some older people believe that talking about death and dying could have negative effects and cause bad events [24]. Furthermore, even though ACP could enable their autonomy to be respected, the patients were sometimes worried that ACP could upset family members [24]. These differences in views and wishes about the discussion on end-of-life issues indicate staffs’ need to be sensitive to and have attunement to the patient’s and family members’ desire and readiness to speak about issues regarding end-of-life. These findings also go in line with results from other studies pointing out that ACP should be seen as a process, not an one-time event, and should be based on discussions from several meetings [12,25] and that the patient’s views on life and death are essential and must be taken into account in the ACP process [12,24].

Arman et al. state that ‘little things have the power to preserve dignity and make patients feel they are valued’ [26]. Our study stressed the significance of small details, which could at first be seen as of little importance. In several interviews, informants underlined the importance of paying attention to these details, such as NH staff doing that little extra for the patient. Managing to do these small things reflected both personal characteristics and suitability for the profession, and showed that staff was engaged and interested in the patient, and respecting the patient as an important and valuable person. Looking at these small details as being part of a unity had a bearing on being safe and secure, according to the family members. In contrast, the informants’ experiences of negative details were associated with expectations of staff not being engaged, competent or committed, and risk of patient’s preferences being ignored in the future care when patient approaching death.

Our results demonstrate, from the family members’ perspective, a perceived lack of physician presence at the NH, and also little awareness of ACP meetings with the physicians taking place. Many informants stated the central role of the nurse and that planned meetings with the physician were not always considered necessary. But at the same time, several informants perceived that they themselves were the ones who were responsible for making decisions regarding the direction of care and treatment limits, and this might be associated with the experience that the physician was described as invisible at the NH. The important role of the nurse is supported by Bollig et al., who showed that trust-based relationships between nurses and the NH patients, as well as between staff and relatives were essential [27]. The nurse’s close relationship with the patient and the family members, in the best-case scenario being familiar with the patient’s values and wishes and having a good rapport with family members, is important, but cannot fully compensate for the absence of the physician. Nurses and physicians have different roles and responsibilities and are not interchangeable in the ACP process.

In Sweden, primary care is divided between two different authorities: regions and municipalities. Each NH has a physician in charge, most often working at a health center as a GP, employed by the region. Nurses working at NHs are municipality employees, which means that physicians and nurses working at NHs have different employers, and that they therefore also use different systems for documenting medical records. As mentioned in the introduction, according to Swedish regulations [14], only physicians can make decisions regarding restrictions of life-sustaining care, not nurses (whose role is to be consulted to aid the decision-making and minimise subjectivity) or the family members [14]. Therefore, when planning care for NH patients, physician competence should not be seen as superfluous and replaceable, but instead as central. In order to plan and deliver care for severely ill patients, physicians are very much needed when balancing risks, benefits, prognostic factors, as well as consultations exploring patient values at end-of-life, informing and anchoring ACPs with patients and family members. Our opinion is that physician presence should be increased at NHs.

## Strengths and limitations

This study involved 18 participants recruited through purposeful sampling [17]. Dependability was reached as audit trail could easily be followed through the whole process [17]. Data was collected from four different NHs, which could be seen as data triangulation [17]. The participants were recruited through maximum variation sampling [17] with the purpose to reach broad variation, which enables transferability [17] to similar settings in NHs. Four different researchers were involved in the analysis of the data, strengthening the results through investigator triangulation [17]. This partly ensured credibility [17]. All four authors were physicians, which could be identified as a limitation. Including a nurse in the research team, may have enriched the analysis process. A GP with experience in the care of NH patients, performed all the interviews, and this could have influenced the answers of the informants. However, all authors were aware of this risk, and the interviewer tried to stay neutral to it during the interviews as well as in the process of the analysis, thus being a neutral investigator [17].

## Implications

The results of this study have implications for staff caring for patients near end-of-life, especially patients living in NHs. Staff involved with these patients should consider raising end-of-life questions with the patient and family members at an early phase, yet should be attuned to the patient’s and family members’ readiness to speak about these issues. Staff should bear in mind that details which could be considered as meaningless may be highly valued from the perspective of family members and that these details often symbolise staff engagement, commitment, competence and attention to the patient’s preferences in care. Family members want to be involved in the ACP process. However, family members need guidance from staff with medical competence when being involved in end-of-life questions and decisions concerning treatment limits, to avoid family members feeling guilt and burden. Health care staff are responsible for the decision-making. Effective ACP implementation, mainly through staff education, has previously been shown to improve NH routines, documentation of preferences, and better adherence to the documents as well as fewer hospital admissions and deaths in hospital [15]. ACP intervention consisting of an education programme to staff in NHs showed increased communication satisfaction from the perspective of both nurses and family members [28]. Good communication with the patients and their family members is fundamental to developing the conditions in which patients near end-of-life can experience autonomy, participation and quality of life, and is important for the family members to feel confident and involved in the care of their loved ones.

The present study has demonstrated new knowledge concerning the ACP process in a NH context from the perspective of family members, which could lead to improvements when implemented. There is a need for further studies on this topic, e.g. studies on documented ACPs in NH patients’ medical records and possible associations between documented ACPs and factors concerning the quality of end-of-life care.
